# Changes of Self-Rated Health Status, Overweight and Physical Activity During Childhood and Adolescence—The Ratchet Effect of High Parental Socioeconomic Status

**DOI:** 10.3389/fspor.2022.781394

**Published:** 2022-03-04

**Authors:** Lea Rittsteiger, Thomas Hinz, Doris Oriwol, Hagen Wäsche, Steffen Schmidt, Simon Kolb, Alexander Woll

**Affiliations:** ^1^Institute of Sports and Sports Science, Karlsruhe Institute of Technology (KIT), Karlsruhe, Germany; ^2^Department of History, Sociology, Empirical Educational Research and Sport Science, University of Konstanz, Konstanz, Germany

**Keywords:** self-rated health status, overweight, physical activity, childhood, adolescence, socioeconomic status, transition rates

## Abstract

Childhood and adolescence are important life periods for the development of health status and physical activity (PA) behaviours. This study analyses the stability and potential changes of self-rated health status, overweight and PA behaviour over time, specifically focusing on the age and the socioeconomic status of children and adolescents. We employ representative longitudinal data for German children and adolescents from the Motorik-Modul Study and the German Health Interview and Examination Survey. Using four different dichotomous health status and PA indicators (self-rated health status [SRHS]; overweight; moderate-to-vigorous PA; and leisure sports engagement), we report *within-person* transition rates across the panel waves when the survey was taken (2003–2006, 2009–2012, and 2014–2017). Additionally, we report results of logistic regressions estimating the impact of children's age, gender, migration background, and their parents' socioeconomic status on these transition rates. The transition rates show mixed results. While children and adolescents from highly problematic states reporting bad SRHS and no leisure sports engagement at an early stage tend to improve later on, overweight children mostly stay overweight. Age and social inequality indicators correlate with some of the chances of improving or worsening the health and PA states. Most clearly, high parental status prevents the health status and PA from worsening over all transitions, particularly becoming overweight, representing a ratchet effect. The results of the present study underline that health policy needs to target specific groups to reduce social inequality in the health status and PA of children and adolescents.

## Introduction

Childhood and adolescence have been shown to be important life periods for the development of behavioural patterns and habits, including health-related behaviours such as regular physical activity (PA) (Viner et al., 2015). At the same time, childhood and adolescence are characterised by continuous development and changes, like puberty or school transitions, which allow and require adaptation to new circumstances (Nelson et al., 2008; Pearson et al., 2017). In this way, new behavioural patterns, like changes in PA or dietary habits, can be formed and established. PA and health in the early stages of life are known to influence PA and health in adulthood (Trudeau et al., 2004; Craigie et al., 2011; Simmonds et al., 2016; Batista et al., 2019). Therefore, it is important to promote an active and healthy lifestyle in the early stages of life to foster health and prevent diseases over the lifespan. Understanding how PA patterns and health track from childhood to adulthood can help reach this goal.

Several literature reviews and meta-analyses have summarised the existing knowledge concerning the stability and changes of PA and health status during childhood and adolescence. To summarise these research results, PA and health status mostly show moderate stability from early childhood to adolescence (Evans et al., 2009; Jones et al., 2013). However, looking at individual studies, the results are somewhat heterogeneous (Jones et al., 2013). Meta-analyses showed a change in PA of −18.1 percent to 7.8 percent from middle childhood to adolescence, with only one study reporting a positive change (Dumith et al., 2011).

For Germany, many studies on tracking PA from childhood to adulthood have used small and non-representative data (Rauner et al., 2015). In a longitudinal study, Rauner et al. (2015) investigated how the tracking of PA during adolescence differentiates by the specific PA setting using a subsample from the Motorik-Modul Study (MoMo). Analysing data from two time periods (2003–2006 and 2009–2012), they found some changes of leisure-time PA in and outside of sports clubs and of overall daily moderate-to-vigorous PA (MVPA) over time. However, tracking correlations were in general low and the study did not investigate the determinants of changes. Schmidt et al. (2020) also used MoMo data and compared PA in curricular sports, extracurricular sports, sports clubs, and unorganised sports in leisure time between two time periods (2003–2006 and 2014–2017). They found that PA in total remains relatively stable, whereas organised sports engagement (in clubs) increased and unorganised sports engagement decreased.

Regarding children's health status and PA, an important aspect to consider is social inequality. Children coming from families with a higher socioeconomic status (SES) resulting from their parents' education, profession, and income evidentially have an advantage regarding health status and PA over children from families with a lower SES. Children with higher parental SES are more physically active (Stalsberg and Pedersen, 2010; Lampert and Kuntz, 2019); do more sports (Mess and Woll, 2012); are less likely to be overweight (Shrewsbury and Wardle, 2008; Barriuso et al., 2015); and are healthier (Reinhold and Jürges, 2012; Kuntz et al., 2018; Lampert and Kuntz, 2019) compared to children with lower parental SES. These differences can, on the one hand, be explained by parents' differing amount of economic capital (Bourdieu, 1992). For example, parents with a higher income can more easily afford sports club memberships or medication for their children than parents with less economic capital. On the other hand, health literacy, i.e. the knowledge about a healthy lifestyle (including regular PA and healthy nutrition), can be considered human capital and is more pronounced in higher social classes (Paasche-Orlow et al., 2005; Stormacq et al., 2019). Better health literacy is associated with better health outcomes (Stormacq et al., 2019). Taken together, children from different social backgrounds have respectively different material and informational resources for a healthy life.

In this study, we will add to the existing literature by systematically tracking changes in PA and health status and differentiating them by parental socio-economic status, gender, and migration background. We exploit a comprehensive and representative data set for Germany which comprises three waves of measurement covering 6 to 12 years of within-person changes. The main focus social status variable will help to gain a better understanding of how the PA and health status of children and adolescents improve or worsen over time. More specifically, we study how children's age and the three indicators of social status (SES, gender, and migration background) impact the *development* of PA and health in children and adolescents. Our research design specifically takes on highly problematic groups among children and adolescents with low PA, overweight, and bad self-reported health and analyses the transition rates into and out these problematic groups. Studying long-term changes and stability of PA and health status with regard to social status will help to develop more targeted policy measures towards a healthy life.

## Data and Methods

### Data

We use data from the MoMo Study, which is part of the German Health Interview and Examination Survey for Children and Adolescents (KiGGS), a nationwide representative study on health, PA, nutrition, and media consumption. The study includes three waves of measurement (T1: 2003–2006; T2: 2009–2012; and T3: 2014–2017) and combines a panel design with a cohort design, i.e. on the one hand participants are interviewed repeatedly and, on the other, a new cohort is added each wave. The MoMo sample was drawn according to three steps. As a first step, 167 sample units were drawn from an inventory of German communities. The sample units were stratified according to their grade of urbanisation (their BIK classification) and their geographic distribution (Kurth et al., 2008). The probability of picking a community was proportional to the number of residents aged under 18 (Schmidt et al., 2020). As the second step, at each sample unit, addresses (*n* = 24) were randomly selected from local population directories (Kurth et al., 2008). As the third step, participants of the overall sample for KiGGS were randomly assigned to the MoMo Study (Schmidt et al., 2019). The pooled dataset combining all three time points included 15,865 observations from 10,404 participants from a very broad range of age. For the transition analysis during childhood and adolescence, we restricted the age of participants at the initial time point (T1 or T2) to 6–17 years, and we only included participants who sequentially took part in more than one survey. This left us with 6,637 observations from 4,045 participants. Variables used for the analyses stem from self-reports of children, adolescents resp. their parents (depending on the age of the children). The transition from T1 to T2 included respondents with both measurements. The same applied to the transition from T2 to T3. Please note that the time interval between two points of measurement is five to six years on average. During data collection, all legal standards on ethics and data privacy were met (Kurth et al., 2008).

### Measures of Health Status

To measure the latent concept of health, research has investigated a variety of health indicators, including cardiovascular health (Lavie et al., 2019), pain or injury (e.g. Myrtveit et al., 2014), and mental health (Lubans et al., 2016). First, we included overweight and adiposity because it has been shown that being overweight increases the risk of several diseases, such as cardiovascular diseases and depression, and negatively impacts health-related quality of life (Dankel et al., 2017). Overweight is measured using the Body Mass Index (BMI), which is recommended by the International Obesity Task Force Childhood Group as well as the European Childhood Obesity Group (Kromeyer-Hauschild et al., 2001). The metric BMI score has been categorised using percentile curves into underweight (> P BMI 18.5), normal weight, overweight (> P BMI 25–P BMI 30), and obese (> P BMI 30) (Cole and Lobstein, 2012). We dichotomized the variable into no overweight (underweight and normal weight) and overweight (overweight and obese).

Second, we included the participants' self-rated health status SRHS because it captures their general, overall health condition. SRHS is measured by self-report using the question “How would you describe your health in general?”, with possible answers being very good, good, fair, bad, and very bad, based on the recommendation of the World Health Organization (Bruin et al., 1996). For children under the age of 11, their parents were asked how they would describe their child's health in general. The answers are dichotomized into bad (very bad–fair) and good (good–very good) SRHS based on the described self-rated health ratings.

### Measures of PA

As for health, there are several ways to assess PA, e.g. using accelerometers (Bornstein et al., 2011) or setting-specific measuring (e.g. Rauner et al., 2015). We included two general measurements of PA: daily MVPA and leisure sports engagement, measured by self-report. The MoMo PA questionnaire was tested and found to have similar reliability and validity to other internationally published PA questionnaires for youth (Jekauc et al., 2012).

Daily MVPA was measured through the answers to the two following questions: “On how many days in the *last seven days* have you been physically active for at least 60 min per day?” and “On how many days are you physically active for at least 60 min in a *typical week*?” Before the questions were asked, PA was defined as activities that make the heart rate and respiration go up, followed by examples for PA (e.g. sports, riding a bicycle) (Schmidt et al., 2016). The answers from both questions were combined and dichotomized into 0–1 days (low MVPA) and 2–7 days (high MVPA).

Leisure sports engagement combines the minutes per week spent doing sports outside school, i.e. in sports clubs and unorganised (outside of clubs) (Schmidt et al., 2016). The respondents with zero minutes of leisure sports engagement (outside school) were assigned to the category of no sports engagement; respondents with at least one minute spent doing leisure sports per week were assigned to the category of sports engagement.

The four variables were constructed to differentiate the group showing highly problematic states of health and unhealthy forms of PA behaviour from the broad (normal) contrast group. As described, we used dichotomized groups of overweight/obese (vs. normal weight/underweight; for the sake of brevity, we phrase the dichotomy as “overweight” vs. “no overweight”), low SRHS (vs. high SRHS), low MVPA (vs. high MVPA), and no leisure sports (vs. at least some leisure sports). This strategy was chosen to analyse the *transitions* out of and into these problematic states over time, i.e. from T1 to T2 and from T2 to T3. In other words: This strategy allows to identify how likely it is to improve or worsen health status and PA at different stages of age.

### Social Status Variables: Age, Parental SES, Gender, and Migration Background

We included children's age at the time of the survey, parental SES, gender, and migration background in our analysis. Age is categorised into 6–10 years, 11–13 years, and 14–17 years at the respective initial state (T1 or T2). Parental SES is a combined measure of the highest education, profession, and income of the household a child lives in. Parents are assigned 1–7 points for each of the three dimensions so that their SES score ranges from 3 to 21. For the categorisation into low, medium, and high SES, the metric SES score was divided into five quintiles. The middle three quintiles are combined to form the category medium SES; the lowest quintile forms the category low SES; and the highest quintile becomes the high SES category. Therefore, the medium SES group contains 60 percent of all observations; the low SES group contains the lowest 20 percent of all observations; and the high SES group contains the highest 20 percent of all observations (Lampert et al., 2018). Migration background includes children with a one-sided and with a two-sided migration background.

### Statistical Analysis

*First*, we start with a brief description of the dataset. We then show the prevalence estimators of problematic health status (SRHS [bad] and overweight) and unhealthy behaviour (MVPA [low] and no leisure sports engagement) at the three time points, while adjusting for the age composition of the surveys. As the second step, we investigate within-person changes of the health and PA indicators by reporting transition rates. The transition rate provide the likelihood of changing/not changing one category of the four dichotomous variables from T1 to T2 and from T2 to T3. As the *third* step, we conduct random effects (RE) logistic regression of the independent variables (age, SES, gender, and migration background) on the health status and PA indicators using the pooled dataset of all three waves (unbalanced panel). This gives overall effects of social status variables on the prevalence estimates. As the *fourth* step, we conduct separate logistic regressions for changing (i.e. improving resp. worsening) rates of the PA and health indicators using the (balanced) panel dataset of two time points respectively (T1 and T2; T2 and T3). Improving denotes the transition from a highly problematic (negative) initial state (bad SHRS, overweight, low MVPA, no leisure sports engagement) to a normal (positive) state (good SRHS, no overweight, normal MVPA, at least some leisure sports engagement). Worsening denotes the transition from a positive initial state to a negative state. To simplify the interpretation of the regression results, we calculated average marginal effects (AME) and their standard errors. The AME can be easily interpreted as the percentage change of the transition rates for the independent grouping variables against the reference category. All analyses were conducted using Stata 14.2.

## Results

Table 1 shows all variables and their categories, including the number of observations (N) and percentages of valid cases (%) in each category. Please note that the pooled data set contains cases from all three waves and the number of cases available for further analysis vary due to selected sub-groups (e.g. transitions from T1 to T2) and missing values (e.g. migration background with a relatively high number of missing values). The main message of Table 1 is that the highly problematic groups of bad SRHS, overweight, low MVPA, and no leisure sports engagement make up about 7 to 18 percent of the respondents. Variables of social status cover a broad range of values—in all dimensions (parental SES, age, gender, migration background).

**Table 1:** Distribution of variables from the working sample (pooled dataset).

**Variable**	**Categories**	**N (total 6,637)**	**% of valid**
Health status groups			
Self-Reported Health-Status (SRHS)			
	Bad/fair	485	7.7
	Good/very good	5,810	92.3
	missing values	342	
Body-Mass-Index (IOTF)			
	Overweight/obese	1,039	17.6
	Normal weight/underweight	4,850	82.7
	missing values	748	
Physical activity groups			
Moderate-to-vigorous physical activity (MVPA)			
	Low (0–1 days)	434	6.8
	High (2–7 days)	5,943	93.2
	missing values	260	
Engage in leisure sports			
	No	1,085	16.8
	Yes	5,358	83.2
	missing values	194	
Social status variables			
Parental Socio-Economic Status (SES)			
	Low	1,319	20.4
	Medium	3,889	60.2
	High	1,245	19.2
	missing values	184	
Age at survey participation (in groups)			
	6–10 years	2,244	33.8
	11–13 years	1,976	29.8
	14–17 years	2,417	36.4
	missing values	0	
Gender			
	Male	3,189	48.1
	Female	3,448	51.9
	missing values	0	
Migration background			
	No	4,761	87.2
	Yes	702	12.8
	missing values	1,174	
Survey participation (wave)			
	T1 (2003–2006)	2,342	35.3
	T2 (2009–2012)	2,889	43.5
	T3 (2014–2017)	1,406	21.2
	missing values	0	

*MoMo/KiGGS; the sample used is restricted to children and adolescents who participated two or three times and who fell into age groups 6–17 years; 6,637 observations stem from 4,045 persons*.

Table 2 provides a cross-sectional overview of prevalence estimation adjusted for the age composition of the three points of measurement (age categories added as control variables in logistic regression models). The prevalence of problematic health status and unhealthy behaviour increases for three of the four variables over time. The comparison of the 95% confidence intervals indicate that bad SRHS significantly grows from 6 percent (T1) to 10 percent (T3). Similarly, overweight is significantly more prevalent (21 percent) at T3 compared to T1 (16 percent). While MVPA does not change over points of measurement the proportion of children and adolescent not engaging in leisure sports at all increases from 16 percent to 20 percent.

**Table 2:** Prevalence estimators of problematic health status and unhealthy behaviour (cross-sectional data, adjusted for age composition).

**Status/behaviour**	**Time of measurement**	**Prevalence estimation**	**95% CI**	**N**
SHRS (bad)	T1 (2003–2006)	6%	5.3–7.4	2,145
	T2 (2009–2012)	8%	6.7–8.6	2,775
	T3 (2014–2017)	10%	8.5–11.7	1,353
Overweight/obese	T1	16%	14.6–17.6	2,334
	T2	17%	15.9–19.0	2,380
	T3	21%	18.7–23.4	1,175
MVPA (low, 0–1 days)	T1	8%	6.5–8.6	2,134
	T2	6%	4.7–6.4	2,865
	T3	8%	6.8–9.7	1,356
No leisure sports	T1	16%	14.6–17.5	2,332
	T2	16%	14.6–17.3	2,783
	T3	20%	18.0–22.3	1,328

**Figure 1:**
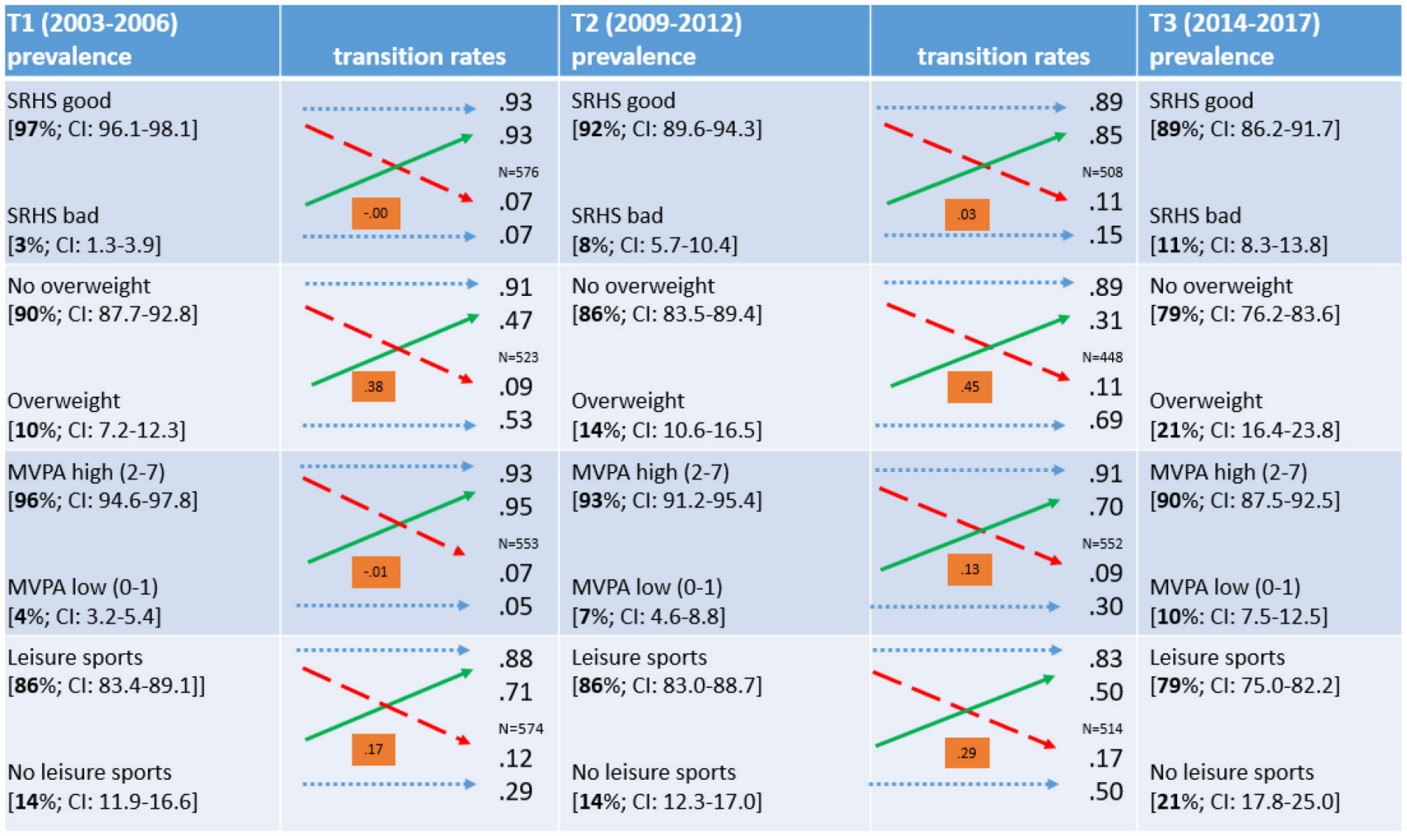
Transitions of the dependent variables between the surveys. MoMo/KiGGS; sample restricted to respondents who participated in all three waves within age of 6–17 years (when having been surveyed); number of cases also vary due to missing values; for prevalence rates 95%- confidence intervals are reported; dotted blue arrows represent stability across times of measurements; dashed red arrows represent transitions from the normal to the problematic group (worsening); solid green arrows represent transitions from the problematic to the normal group (improving); for each transition table Kendall's Tau b reported as an indicator of strength of the association (range of value from −1 to +1). An association indicates whether the distribution of participants into dichotomous categories at an earlier time of measurement has a notable predictive power for the distribution at the subsequent measurement.

In order to combine prevalence and transitions rates and to make their comparisons straightforward, we restricted the working sample to those study participants who took part in *all three* MoMo waves. This sample restriction changes the prevalence rates reported above slightly as Figure 1 depicts. At T1 and T2, there are lower prevalence rates compared to the cross-sectional analysis—most probably due to positive selection processes to participate in repeated surveys. Again, the prevalence rates of the restricted sample at T1, T2, and T3 are given in the respective columns (values in percent are given in square brackets with 95% confidence intervals). Comparing the first and the second time point, we see—for instance—an increase in the prevalence of the problematic states and behaviours for three of four variables. Bad SRHS increased from 3 to 8 percent; overweight increased from 10 to 14 percent; low MVPA increased from 4 to 7 percent; and no leisure sports engagement remains stable at 14 percent. Comparing the second and third survey, we see an even slightly higher increase in the prevalence of the problematic groups. Looking at both the cross-sectional analysis and the restricted analysis of prevalence rates, one might conclude an overall deterioration across all four variables over time, specifically between T1 and T3.

Figure 1 also illustrates for the restricted sample at hand the *transitions* of observations of the dependent variables between the first and the second, as well as between the second and the third, survey. The blue dotted arrows indicate stability between points of measurement, green solid arrow depict improvements while red dashed arrows show changes to the worse. The transition rates are denoted next to the arrows can be interpreted as improving and worsening rates. For example, of the 10 percent of respondents with overweight in the first survey, 47 percent (0.47) improved to normal weight in the second survey. Of the 90 percent of respondents with normal weight in the first survey, 9 percent (0.09) had overweight in the second survey. In addition, Figure 1 contains a coefficient of association of the dichotomous variables at the two transitions (Kendall's tau b). The coefficients intuitively inform about how state of origin impacts the state of destination. For instance, the coefficients for the transitions of overweight are much higher the coefficients for the SRHS transitions, i.e. being overweight at T1 has a higher predictive information value for being overweight at T2, in comparison to the SRHS categories. Summarising the transition rates, SRHS, MVPA, and sports engagement show remarkable improvement over time. An improvement in overweight is visible, although the majority of overweight children stayed overweight across the three waves. Given positive states for all four indicators at the beginning of the process, all indicators show relatively low worsening rates.

Taking the descriptive picture of the prevalence and transition rates together, we see that the overall increase in the prevalence of problematic states of health and unhealthy behaviour is due to these low worsening rates of the majority, while the minority in the problematic states have some chance of improving. Comparing the first transition (T1–T2) to the second transition (T2–T3), we find rather similar results. Therefore, for the regression analysis, the two transitions are combined.

The estimates of the logistic regression models (reporting AME and standard errors) are depicted in Tables 3, 4. Table 3 shows the results of the RE logistic regression, pooling all measurements from three time points. Coefficients can be interpreted as percentage changes in the probability of being in each of the *less* problematic states at all points of measurement (SRHS: good; no overweight; MVPA: at least some; Sports: at least some). In other words, this step in the analysis informs about how prevalence rates change with covariates. The variable Survey *Participation* indicates the wave in which an observation was measured. Reporting a SRHS is clearly affected by age groups: In the age group 11–13 years, the prevalence rate of a good SRHS is 7 percentage points less (compared to 6–10 years). In contrast, parental SES has a positive impact: the high SES group has 6 percentage point higher probability of a good SRHS. According to the results in the second column of Table 3, children and adolescents with higher parental SES are more likely to have no overweight. To put it differently: They are less likely to be overweight. Compared to the first survey, measurements from the second and the third survey reveal a slightly lower tendency of being normal weight. If one controls the social status variables there seems to be a slight trend to overweight over time.

**Table 3:** Logistic regression of (positive) health status and (positive) PA indicators on social status variables (Random effects; average marginal effects).

		**SRHS (good)**	**No overweight**	**MVPA (high)**	**Leisure sports (yes)**
Age (Ref: 6–10)	11–13	−0.07^***^	−0.02	−0.03^***^	0.02
		(0.016)	(0.012)	(0.008)	(0.012)
	14–17	−0.05^***^	0.001	−0.07^***^	−0.03^*^
		(0.013)	(0.012)	(0.009)	(0.013)
Parental SES (Ref: Low)	Medium	0.03^**^	0.03^*^	0.02^*^	0.08^***^
		(0.010)	(0.014)	(0.009)	(0.015)
	High	0.06^***^	0.08^***^	0.02	0.16^***^
		(0.015)	(0.017)	(0.012)	(0.016)
Gender (Ref: Male)	Female	−0.006	0.004	−0.04^***^	−0.05^***^
		(0.007)	(0.011)	(0.007)	(0.011)
Migration background (Ref: No)	Yes	−0.02^*^	−0.005	−0.01	−0.05^***^
		(0.010)	(0.016)	(0.010)	(0.014)
Participation: Survey (Ref. T1)	T2	0.001	−0.02^*^	0.03^***^	−0.003
		(0.008)	(0.010)	(0.008)	(0.010)
	T3	0.01	−0.06^*^	0.03	−0.02
		(0.016)	(0.027)	(0.015)	(0.025)
N		4,971	4,779	5,056	5,164

*MoMo/KiGGS, working sample, pooled data from T1, T2, and T3*.

* *p < 0.10,*

** *p < 0.05,*

*** *p < 0.001; standard errors in parentheses; random effects models consider the error terms to be nested within persons; average marginal effects (AME) estimate the change in prevalence rates against the reference category of independent variables*.

**Table 4:** Logistic regression of the health status and PA indicators, differentiated by improving and worsening (average marginal effects).

		**SRHS**		**No overweight**		**MVPA**		**Sports**	
		**Improving**	**Worsening**	**Improving**	**Worsening**	**Improving**	**Worsening**	**Improving**	**Worsening**
Age (Ref: 6–10)	11–13	0.00	0.01	0.04	0.01	−0.01	0.04^***^	−0.11	0.05^**^
		(0.06)	(0.01)	(0.06)	(0.02)	(0.06)	(0.01)	(0.05)	(0.02)
	14–17	−0.14^*^	−0.01	−0.10	0.04	−0.10	0.06^***^	−0.03	0.02
		(0.07)	(0.01)	(0.07)	(0.02)	(0.06)	(0.01)	(0.05)	(0.02)
Parental SES (Ref: Low)	Medium	−0.14^**^	−0.03^*^	0.04	−0.05^*^	0.06	−0.02	0.10^*^	−0.09^***^
		(0.05)	(0.01)	(0.06)	(0.02)	(0.07)	(0.01)	(0.05)	(0.02)
	High	−0.13	−0.04^*^	0.09	−0.09^***^	0.18^*^	−0.03^*^	0.08	−0.13^***^
		(0.09)	(0.02)	(0.09)	(0.02)	(0.07)	(0.02)	(0.08)	(0.02)
Gender (Ref: Male)	Female	−0.13^*^	0.02^*^	0.03	−0.03^*^	−0.02	0.04^***^	0.03	0.05^***^
		(0.05)	(0.01)	(0.05)	(0.01)	(0.06)	(0.01)	(0.04)	(0.01)
Migration background (Ref: No)	Yes	−0.23^**^	0.02	−0.01	0.02	−0.01	−0.00	−0.04	0.01
		(0.08)	(0.02)	(0.08)	(0.02)	(0.08)	(0.01)	(0.06)	(0.02)
N at risk		223	3,411	338	1,904	221	3,471	540	3,155

*MoMo/KiGGS, pooled data for transitions from T1 to T2, and from T2 to T3*.

* *p < 0.10,*

** *p < 0.05,*

*** *p < 0.001 standard errors in parentheses*.

Older respondents and females are less likely to be physically active on more than one day of the week (MVPA, see third column of Table 3). Compared to the first survey, measurements from the second survey now reveal a slightly higher (normal) activity, whereas measurements from the third survey are not significantly different from those of the first survey. Participants with medium and high parental SES are more likely to actively engage in sports compared to participants with low parental SES, whereas females and participants with a migration background are less likely to engage in leisure sports. So far, the analysis reveals interesting and consistent patterns of health status and PA with social status variables. Parental SES positively correlates with three of four indicators for good health status and healthy behaviour while girls are less active in PA, and participants at a higher age (11–13, and 14–17) tend to report lower SRHS and to be less active, specifically regarding MVPA.

The final step of analysis will show if the coefficients differ between respondents who improve and respondents who worsen their behaviour. The working sample now contains all participants who sequentially took part at least twice. Because transition rates were rather similar for both transitions, data have been pooled for transitions T1 to T2 and T2 to T3—as already mentioned, for both directions of change—improving and worsening—Table 4 shows that *age* mainly affects the PA indicators in a negative way, and barely affects the health indicators. Older children are more likely to worsen their MVPA and sports engagement. The oldest age group (aged 14–17) is less likely to improve its SRHS compared to the youngest age group (aged 6–10). Parental *SES* affects both health and PA indicators. Compared to children and adolescents with a low parental SES, children and adolescents with higher parental SES are less likely to improve their SRHS, but also less likely to worsen it; therefore, their SRHS is more stable. Children and adolescents with a high parental SES are also less likely to worsen from no overweight to overweight. Regarding the PA indicators, they are more likely to improve and less likely to worsen their MVPA, and less likely to worsen their leisure sports engagement. Overall, parental SES shows positive health effects, mainly by reducing the likelihood of worsening PA and health status indicators.

There are differentiating trends by *gender*. Girls are more likely to worsen their MVPA and sports engagement than boys—i.e. girls who are physically active are more likely to lower their PA over time than boys. However, girls who are *not* physically active are *not* less likely to improve their PA than boys. Finally, *migration background* only correlates with SRHS. Children and adolescents with a migration background are less likely to improve their SRHS compared to their peers without a migration background.

## Discussion

The results of the present study show that children and adolescents with highly problematic health and PA states have rather good chances to improve later on, with the clear exception of being overweight. If children are overweight at their first measurement, they stay overweight with a probability of about 53 percent, and if children are overweight at their second measurement, they stay overweight with an even higher probability of about 69 percent. At the same time, children and adolescents who show good health and PA states at earlier stages have a low transition rate to the highly problematic health groups (from 7 to 17 percent). The consistently and often-reported research results on the average decline of PA during adolescence (Evans et al., 2009; Dumith et al., 2011) are the result of these two counteracting transition processes. Taken together, the growing decline of PA for adolescents (on average) mainly feeds out of a small transition rate of the larger group being in “normal” states earlier, while SRHS and sports engagement improve for the smaller (highly problematic) group. In our transitory analysis, we found only small negative effects of higher age on sports engagement (MVPA). This is in line with a recent study, also using MoMo data, in which Schmidt et al. (2020) found that PA remained fairly stable among children and adolescents as well.

In contrast to the SRHS and PA indicators, overweight is mostly stable over time, i.e. the transition rate out of being overweight is relatively small, at least the smallest given all other transitions under study. In other words, children and adolescents who are overweight have a significant likelihood to stay overweight in the second and third measurements. This is in line with the results of Schienkiewitz et al. (2018), who compare children's overweight in the first and second wave of KiGGS, as well as Spengler et al. (2014), who even show an increase in overweight over time using MoMo data. High parental SES has a double impact: it negatively affects the chances of being in the highly problematic group (in the early stages) and causes the transition rates to worsen across the measurements. Thus, high parental SES works—to a certain extent—as a factor preventing the development of overweight during childhood and adolescence, while losing the status of overweight is not affected by parental SES. Thus, high parental SES literally has a ratchet effect.

When investigating the correlation of age with health and the PA of children and adolescents in Germany, the German school system should be considered. From the ages of 6 to 10, children in Germany typically go to primary school (Grades 1–4). In Grade 4, children and their parents must decide on a secondary school type based on the child's grades and abilities (Wiedenhorn, 2011). The transition from primary to secondary school is accompanied by major changes in children's lives—e.g. longer hours of schooling and longer commutes, new subjects, new friends, and new teachers (Harazd and Schürer, 2006)—which influence children's well-being and PA. Krampen (2013) reports significant discontinuities in German children's subjective well-being, e.g. negative changes before and after educational transitions. In accordance with Pearson et al. (2017), who found that educational transitions lead to an increase in sedentary time, we detected a reduction in sports engagement for older age groups (11–13 and 14–17). In particular, the likelihood of the MVPA worsening is higher for these groups compared to children in primary school.

Regarding the research question of how the measures of social inequality influence the development of health status and PA of children and adolescents, we see that the different indicators of social inequality influence specific indicators of PA and health status. Controlling for parental SES, migration background was mainly irrelevant—with the exception of the chances of improving low SRHS. As already discussed, parental SES mainly correlates with being overweight, while gender is relevant for sports engagement and its changes. In this respect, our analysis reveals differentiated results. Girls with good PA measurements are more likely to worsen their PA engagement over time than boys, while girls with bad PA are not more or less likely to improve their PA than boys. This can be used to take measures that specifically target groups at risk of not improving or worsening. For example, to support girls' PA, measures must not only target girls with bad PA and encourage them to improve their engagement; they must also target girls with good PA and encourage them *not* to worsen their engagement.

### Limitations

Although we used informative longitudinal (high-quality) data for a representative sample of German children and adolescents, the present study is subject to some limitations. The first limitation is the assessment of the dependent variables SRHS, MVPA, and sports engagement by *self-reporting*, which is particularly difficult for young children. However, all children under the age of 11 were accompanied by a parent or other responsible adult who could check and confirm their answers or help with assessing time frames (Bös et al., 2009). Self-reporting methods have limitations, such as recall bias and biassed answers due to social desirability (Adams et al., 2005). For instance, the latter might explain why an overwhelming majority of respondents reported at least a good SRHS (Camerini and Schulz, 2018). However, objective measures of PA like accelerometers or pedometers also have severe limitations, such as short time intervals for measuring PA. Furthermore, they do not account for seasonality effects. Therefore, we assume that self-reporting is an adequate and sufficiently reliable source to measure habitual MVPA and usual sports engagement.

In more detail, SRHS was not reported by children under the age of 11, but instead by their parents, which might lead to skewed results for the age group of 6 to 10-year olds. For children aged 11 and above, we have observations from children and from parents reporting on the child's SRHS. In the overwhelming majority of cases, the child's response did not exactly match the parent's response, and the children themselves tended to report a worse SRHS than their parents. For children under the age of 11, we can therefore assume that their actual SRHS is worse than what was shown in our results.

A further aspect concerning two of the dependent variables might be that MVPA and sports engagement overlap to some degree, since both measure habitual PA. However, we purposely differentiated between the two, first to investigate whether there were differences between daily *general PA* (which for instance also includes walking to the train station) and PA that is specifically perceived as *doing leisure sports outside school*; and second, the two indicators were measured differently (days vs. minutes) because we assumed this might yield different results. Robustness checks were carried out by using different categorizations of MVPA (0–4 and 5–7 days) and leisure sports (split at the mean). The regression analysis led to similar results (not shown). Comparing both habitual PA variables, MVPA categorises a much more problematic group of respondents as being *physically inactive* beyond the decision to engage consciously in leisure sports.

The second general limitation of the present study is its lack of causal inference. We simply cannot manipulate the independent variables (parental SES; gender; migration background), for obvious reasons. In addition, these variables usually do not change for a person over time. Even though we have analysed time-specific processes, we do not intend to draw a causal conclusion from the covariates under study. Further research is needed to address why we find the ratchet effect of parental SES. So far, we could speculate about possible “class” differences regarding nutrition, actively doing sports, and other aspects of habitual lifestyle.

Lastly, we must consider the time between the three waves and its influence on our analysis. The three surveys were conducted from 2003–2006 (T1), 2009–2012 (T2), and 2014–2017 (T3). As already mentioned, the mean time between the first and the second survey for the respondents was six years (a range of 6–7 years). Between the second and the third survey, the time was a bit shorter −4.8 years (a range of 4–8 years). Between all three surveys, political and social changes occurred. For example, in 2008, the IN FORM initiative of the German Federal Ministry of Food and Agriculture and the Federal Ministry of Health started, which led to changes in health awareness, as well as in health behaviour (Bundesministerium für Ernährung und Landwirtschaft (BMEL), 2019). We cannot rule out that these programmes impacted our respondents' health and PA independently from the influence of our independent variables.

## Conclusion

The present study reveals complex longitudinal changes in health status and PA behaviour during childhood and adolescence. Different social groups of children and adolescents have different likelihoods of improving or worsening their health status and PA. In this context, we found that high parental SES is accompanied by a ratchet effect, particularly with regard to overweight. Groups with a lower parental SES are at higher risk of worsening their health behaviour. Therefore, policy measures must target specific groups to consider the influence of social status indicators on health status and PA and to promote an active and healthy lifestyle. Taken together, the present study indicates that all four variables of health status and PA, and even more importantly, most transitions of improving and worsening, are correlated in a specific manner with social status, even beyond parental SES. Particularly, there seems to be a need for age and gender-specific programs to address the worsening of PA during adolescence.

## Data Availability Statement

The complete dataset cannot be made publicly available because informed consent from study participants did not cover public deposition of data. However, the restricted dataset underlying the reported findings and the syntax files (Stata 14.2) are archived at the Institute of Sports and Sports Science of the Karlsruhe Institute of Technology (KIT) and can be accessed by interested researchers on-site. Enquiries regarding on-site access to the restricted dataset and syntax files should be submitted to Prof. Dr. Alexander Woll, Karlsruhe Institute of Technology, Engler-Bunte-Ring 15, 76131 Karlsruhe, Germany (alexander.woll@kit.edu).

## Ethics Statement

Ethical review and approval was not required for the study on human participants in accordance with the local legislation and institutional requirements. Written informed consent to participate in this study was provided by the participants' legal guardian/next of kin.

## Author Contributions

LR conducted the data analysis and was mainly responsible for writing the article. TH developed the analytical design and contributed to writing. DO, HW, SS, and SK compiled and pre-processed the data and commented on earlier versions of the paper. AW (PI) successfully applied for funding for this research and contributed to writing. All authors contributed to the article and approved the submitted version.

## Funding

This work has been developed using data from the MoMo Study (2009–2021), Physical fitness and physical activity as determinants of health development in children and adolescents. The MoMo Study is funded by the Federal Ministry of Education and Research (Funding Reference Number: 01ER1503). TH was supported by EXC2035, The Politics of Inequality, at the University of Konstanz.

## Conflict of Interest

The authors declare that the research was conducted in the absence of any commercial or financial relationships that could be construed as a potential conflict of interest.

## Publisher's Note

All claims expressed in this article are solely those of the authors and do not necessarily represent those of their affiliated organizations, or those of the publisher, the editors and the reviewers. Any product that may be evaluated in this article, or claim that may be made by its manufacturer, is not guaranteed or endorsed by the publisher.

## Abbreviations

AME: average marginal effects
BMI: Body Mass Index
IOTF: International Obesity Task Force
KiGGS: Health Interview and Examination Survey for Children and Adolescents
MoMo: Motorik-Modul Study
MVPA: moderate-to-vigorous physical activity
PA: physical activity
RE: random effects
SES: Socioeconomic status
SRHS: self-rated health status.
